# Incremental prognostic value of the fibrinogen−to−albumin ratio for adverse perinatal outcomes in preeclampsia: a dual−center retrospective cohort study

**DOI:** 10.3389/fendo.2026.1853375

**Published:** 2026-05-29

**Authors:** Xiaoyun Chen, Zhihong Wang, Yueqiong Wang, Huaijian Zhang, Yahui Liu, Yichao Pan, Ling Chen, Xun Zhang, Jiabin Li, Ye Huang

**Affiliations:** 1 Department of Anesthesiology, Zhangzhou Affiliated Hospital of Fujian Medical University, Zhangzhou, Fujian, China; 2 Department of Neonatology, Zhangzhou Affiliated Hospital of Fujian Medical University, Zhangzhou, Fujian, China; 3 Department of Anesthesiology, The First Affiliated Hospital of Xiamen University, School of Medicine, Xiamen University, Xiamen, Fujian, China; 4 Department of Cardiac Surgery, Fujian Medical University Union Hospital, Fuzhou, Fujian, China

**Keywords:** adverse perinatal outcome, albumin, fibrinogen, incremental value, nomogram, prediction model, preeclampsia

## Abstract

**Objective:**

This study aims to evaluate whether the fibrinogen−to−albumin ratio (FAR) provides incremental prognostic value for identifying preeclamptic patients at high risk of adverse peripartum events, defined as a composite adverse perinatal outcome (CAPO) that serves as a surrogate for severe disease burden, and to explore its potential utility in guiding anesthesia−relevant decisions.

**Methods:**

This dual−center retrospective cohort study included 924 preeclamptic patients from two tertiary referral hospitals. Patients from center A (*n* = 680) were randomly allocated in a 7:3 ratio to a training cohort (*n* = 476) and an internal validation cohort (*n* = 204). Patients from center B (*n* = 244) constituted an independent external validation cohort. CAPO was defined as the occurrence of placental abruption, preterm birth, fetal growth restriction, fetal distress, neonatal respiratory distress syndrome, 5−min Apgar score below 7, neonatal intensive care unit admission, or perinatal death. Candidate predictors were identified using forward stepwise logistic regression. Model A (the base model) incorporated established predictors including the sFlt−1/PlGF ratio. Model B (the extended model) was developed by adding FAR. Model performance was assessed using the area under the receiver operating characteristic curve (AUC), calibration plots, and Brier scores. Incremental value was quantified by the change in AUC (ΔAUC), continuous net reclassification improvement (NRI), and integrated discrimination improvement (IDI).

**Results:**

In the training cohort, model A achieved an AUC of 0.852 (95% confidence interval [CI] 0.818–0.887), whereas model B demonstrated significantly improved discrimination with an AUC of 0.888 (95% CI 0.859–0.917; *P* < 0.001). Model B also showed favorable calibration (Hosmer–Lemeshow, *P* = 0.594) and a lower Brier score (0.134 vs. 0.153). At the optimal cutoff value of 0.135 for FAR (determined by the Youden index), model B achieved a sensitivity of 0.864 and a specificity of 0.740 for predicting CAPO. In the external validation cohort, model B maintained robust discrimination (AUC 0.856, 95% CI 0.808–0.904) and outperformed model A (AUC 0.798, 95% CI 0.740–0.857; *P* = 0.003). Across the entire cohort, the addition of FAR resulted in a ΔAUC of 0.010 (95% CI 0.002–0.019; *P* = 0.013), a continuous NRI of 0.397 (95% CI 0.268–0.526; *P* < 0.001), and an IDI of 0.022 (95% CI 0.012–0.032; *P* = 0.004). Subgroup and sensitivity analyses confirmed the robustness of these findings.

**Conclusions:**

FAR provides statistically significant incremental prognostic value for predicting CAPO, a composite of severe peripartum events. From an anesthesiology perspective, FAR ≥0.135 alerts to coagulopathy and systemic inflammation, supporting cautious neuraxial anesthesia, early invasive monitoring, and escalated perioperative care.

## 1 Introduction

Preeclampsia affects approximately 5%–7% of pregnancies worldwide and accounts for approximately 10 million cases annually (1–4). It remains a leading cause of maternal and perinatal morbidity and mortality, contributing to an estimated 16% of global maternal deaths (5–7). Beyond immediate peripartum complications, preeclampsia is associated with long-term cardiovascular sequelae for the mother and neurodevelopmental delays for the offspring (8–13). Among women diagnosed with preeclampsia, the clinical course is highly heterogeneous: some patients experience relatively benign outcomes, whereas others progress rapidly to life-threatening complications such as placental abruption, eclampsia, HELLP syndrome, or acute kidney injury (14–18). Consequently, accurate risk stratification at the time of diagnosis is essential. It helps guide decisions regarding the intensity of monitoring, timing of delivery, and level of care. From an anesthesiologist’s perspective, preeclampsia presents unique perioperative challenges. These include thrombocytopenia (which increases the risk of neuraxial hematoma), airway edema (which complicates intubation), and hemodynamic instability (which requires precise vasopressor and fluid management). However, no simple, routinely available biomarker currently exists to help anesthesiologists rapidly assess coagulation status, inflammatory burden, and periprocedural risk in preeclamptic patients.

Several multivariable prediction models have been developed to identify preeclamptic patients at elevated risk of adverse outcomes. The fullPIERS model, derived from a large prospective multicenter cohort, predicts adverse maternal outcomes within 48 h to 7 days after admission, incorporating gestational age, chest pain or dyspnea, oxygen saturation, platelet count, serum creatinine, and aspartate aminotransferase (19–21). The PREP-L model was designed specifically for women with early-onset preeclampsia to estimate the overall risk of maternal complications by discharge (22–24). Although both models have demonstrated good discriminatory performance in their respective development and validation cohorts, their primary focus is on maternal rather than perinatal complications (25, 26). Moreover, the performance of fullPIERS in predicting perinatal outcomes has been limited, underscoring the need for prognostic tools that encompass both maternal and fetal/neonatal endpoints.

The soluble fms−like tyrosine kinase−1 to placental growth factor ratio (sFlt−1/PlGF) is currently regarded as the reference biomarker for the short−term prediction and diagnosis of preeclampsia (27). A ratio of ≤38 effectively rules out preeclampsia within 1 week with a negative predictive value exceeding 98%, whereas a ratio ≥85 supports the diagnosis (28, 29). The sFlt−1/PlGF ratio reflects the angiogenic imbalance central to the pathogenesis of preeclampsia and has been incorporated into clinical practice guidelines internationally (27, 30, 31). Nevertheless, its utility is constrained by cost, limited availability in resource−constrained settings, and modest positive predictive value for adverse perinatal outcomes. Furthermore, the incremental benefit of adding sFlt−1/PlGF to established clinical prediction models has been variable across studies, suggesting that complementary biomarkers may further optimize prognostic accuracy.

The fibrinogen−to−albumin ratio (FAR) has recently emerged as a candidate prognostic marker in preeclampsia (32). Fibrinogen is an acute−phase reactant that rises during systemic inflammation, whereas albumin is a negative acute−phase protein that declines in inflammatory states. FAR therefore integrates both pro−inflammatory and pro−coagulant signals and may reflect the severity of the underlying disease process. Preliminary evidence indicates that elevated FAR is associated with an increased risk of adverse perinatal events in preeclamptic patients (33, 34). However, existing studies have primarily evaluated FAR as a predictor of preeclampsia development rather than as a prognostic marker after preeclampsia has been established. Critically, no study has assessed whether FAR provides incremental prognostic value beyond that of the sFlt−1/PlGF ratio in a well−characterized cohort of patients with established preeclampsia.

The objective of this dual−center retrospective cohort study was therefore threefold, namely: first is to develop and validate a multivariable prediction model for composite adverse perinatal outcome (CAPO) in patients with established preeclampsia, incorporating routine clinical and laboratory parameters including the sFlt−1/PlGF ratio, second is to quantify the incremental prognostic value of adding FAR to this model using established statistical metrics of reclassification and discrimination improvement, and third is to explore the implications of FAR for anesthesia-related decision−making.

## 2 Materials and methods

### 2.1 Study design and participants

This dual−center retrospective cohort study was conducted using data from two tertiary referral hospitals (center A and center B) between January 2018 and December 2024. The study protocol was approved by the Institutional Review Board of each participating center, and the requirement for informed consent was waived owing to the retrospective use of de−identified patient data. Eligible patients were identified by screening electronic medical records for a diagnosis of preeclampsia according to the American College of Obstetricians and Gynecologists (ACOG) criteria. The inclusion criteria were as follows: age ≥18 years, singleton pregnancy, admission with a confirmed diagnosis of preeclampsia, and availability of laboratory data obtained within 48 h of admission. Patients were excluded if they had any of the following: pre−existing renal, hepatic, or autoimmune disease documented in the medical record prior to pregnancy or during the first trimester; chronic hypertension predating pregnancy confirmed by pre−pregnancy or first−trimester records; clinical evidence of infection at admission, defined as body temperature >38.0 °C, white blood cell count >15 × 10^9^/L, or a documented diagnosis of infection; or if more than 20% of the predefined study variables were missing from the medical record. *De novo* laboratory abnormalities consistent with preeclampsia−related organ injury were not considered grounds for exclusion.

**Figure 1:**
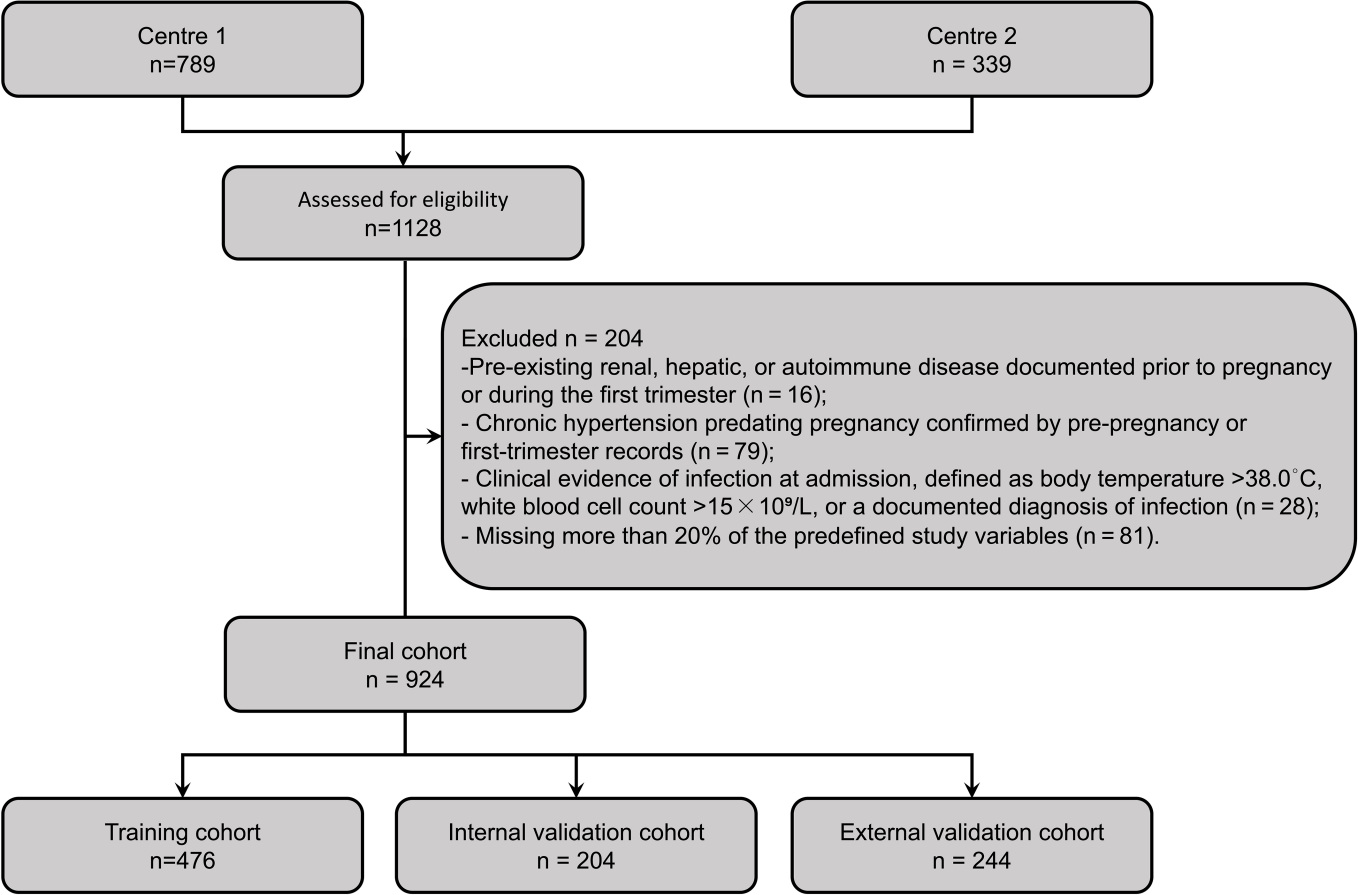
Patient enrollment flowchart and cohort allocation. A total of 1, 128 patients with preeclampsia from two tertiary referral hospitals were screened for eligibility. Following the application of the prespecified exclusion criteria, 204 patients were excluded for the reasons detailed in the flowchart. The final cohort comprised 924 eligible patients. Patients from center 1 (*n* = 680) were randomly assigned in a 7:3 ratio to a training cohort (*n* = 476) for model development and an internal validation cohort (*n* = 204). An additional 244 eligible patients from center 2 constituted the independent external validation cohort.

A total of 1, 128 patients were initially screened, comprising 789 patients from center A and 339 from center B. After application of the exclusion criteria, 680 eligible patients from center A were randomly assigned in a 7:3 ratio to a training cohort (*n* = 476) and an internal validation cohort (*n* = 204). The remaining 244 eligible patients from center B constituted an independent external validation cohort. All laboratory measurements were obtained at admission or within 48 h prior to admission and, crucially, before delivery and the occurrence of any component of the composite outcome. A flow diagram illustrating patient enrollment and cohort allocation is provided in **Figure 1**.

### 2.2 Data collection and definitions

Data were extracted from the electronic medical record systems of the two participating centers. The following categories of information were collected for each eligible patient: demographic characteristics, clinical parameters at admission, laboratory findings obtained within 48 h of admission, and perinatal and maternal outcomes.

The demographic and clinical variables included age, pre−pregnancy body mass index (BMI), gravidity, parity (categorized as nulliparous or multiparous), history of preeclampsia, chronic hypertension, diabetes mellitus (pregestational or gestational), smoking history, gestational age at diagnosis, systolic and diastolic blood pressure, and mean arterial pressure. The mean arterial pressure was calculated as diastolic blood pressure plus one−third of the pulse pressure.

Preeclampsia was diagnosed according to the American College of Obstetricians and Gynecologists (ACOG) 2020 criteria (35), defined as new−onset hypertension (systolic blood pressure ≥140 mmHg or diastolic blood pressure ≥90 mmHg) after 20 weeks of gestation accompanied by proteinuria or evidence of end−organ dysfunction. The diagnoses were verified by review of the treating clinician’s documentation. Disease onset was classified as early−onset (diagnosis before 34 weeks of gestation) or late−onset (diagnosis at or after 34 weeks). Severity was categorized as mild or severe; severe preeclampsia was defined by systolic blood pressure ≥160 mmHg, diastolic blood pressure ≥110 mmHg, or the presence of thrombocytopenia (platelet count <100 × 10^9^/L), renal insufficiency (serum creatinine >1.1 mg/dL or doubling of baseline), impaired liver function (transaminases elevated to twice the upper limit of normal), pulmonary edema, or new−onset headache or visual disturbances as documented in the medical record. Fetal growth restriction (FGR) was defined as a birth weight below the 10th percentile for gestational age according to Chinese neonatal birth weight standards. For cases without a recorded birth weight, an ultrasound−estimated fetal weight below the 10th percentile was used.

Laboratory parameters measured within 48 h of admission included fibrinogen, albumin, platelet count, hemoglobin, creatinine, uric acid, alanine aminotransferase (ALT), aspartate aminotransferase (AST), lactate dehydrogenase (LDH), D−dimer, and the sFlt−1/PlGF ratio. The fibrinogen−to−albumin ratio (FAR) was calculated as fibrinogen (g/L) divided by albumin (g/L). Urine protein excretion was recorded as grams per 24 h. Baseline perfusion index was obtained from pulse oximetry recordings at admission when available.

Delivery- and anesthesia−related variables included mode of delivery (vaginal or cesarean), indication for cesarean section (if applicable), labor induction, and type of anesthesia (epidural, combined spinal–epidural, general, or none). Additional anesthesia−specific variables were recorded: use of invasive arterial blood pressure monitoring, central venous catheter placement, intraoperative blood transfusion, and postoperative analgesic modality (e.g., patient−controlled epidural analgesia or intravenous opioids). These variables were collected to explore the potential role of FAR in guiding perioperative anesthesia decisions.

All predictor variables were ascertained from measurements obtained prior to delivery. The timing of each laboratory measurement and clinical assessment was verified against the delivery timestamp in the electronic medical record to ensure that exposure preceded the occurrence of any component of the composite outcome.

### 2.3 Outcome definition

The primary outcome was composite adverse perinatal outcome (CAPO), defined as the occurrence of any of the following events during the peripartum period: placental abruption, preterm birth, fetal growth restriction (FGR), fetal distress, neonatal respiratory distress syndrome (NRDS), a 5− min Apgar score below 7, admission to the neonatal intensive care unit (NICU), or perinatal death. CAPO was constructed as an integrated endpoint that combines adverse events sharing a common upstream pathophysiology, namely, uteroplacental insufficiency and maternal vascular dysfunction. Placental abruption was included because it arises from the same uteroplacental pathology that underlies preterm birth, FGR, and fetal distress.

Placental abruption was identified by clinical diagnosis—characterized by abdominal pain, vaginal bleeding, or uterine hypertonicity—and was confirmed by placental pathology when available. Preterm birth was defined as delivery before 37 completed weeks of gestation. Fetal growth restriction was defined as previously described (see “Data collection and definitions”). Fetal distress was defined by the presence of an abnormal intrapartum cardiotocographic tracing necessitating expedited delivery, an umbilical arterial pH below 7.20, or a documented clinical diagnosis of fetal distress in the medical record. Neonatal respiratory distress syndrome was diagnosed by the attending neonatologist and required any form of respiratory support. A 5− min Apgar score below 7 was abstracted from the neonatal assessment record. NICU admission was recorded irrespective of the underlying indication. Perinatal death encompassed both stillbirth (fetal death occurring at or after 22 completed weeks of gestation) and neonatal death occurring within the first 7 completed days of life.

Secondary maternal outcomes included postpartum hemorrhage (defined as estimated blood loss ≥500 mL for vaginal delivery or ≥1, 000 mL for cesarean section, occurring within 24 h of birth), eclampsia (defined as new−onset, generalized tonic–clonic seizures occurring in a patient with established preeclampsia, after exclusion of other potential etiologies such as epilepsy, intracranial hemorrhage, cerebral infection, or metabolic encephalopathy), HELLP syndrome (hemolysis, elevated liver enzymes, and thrombocytopenia), acute kidney injury (defined according to the Kidney Disease: Improving Global Outcomes [KDIGO] criteria (36), stage 1 or higher, based primarily on changes in serum creatinine), and a composite of any of the aforementioned severe maternal complications.

All outcomes were ascertained by review of the electronic medical record and, where applicable, cross−referenced with discharge summaries and procedure notes. The composite outcome CAPO was coded as positive if any of the eight individual components occurred and negative only if none of the components were documented.

### 2.4 Statistical analysis

All analyses were performed using R version 4.4.0 (R Foundation for Statistical Computing, Vienna, Austria) and SPSS version 26.0 (IBM Corp., Armonk, NY, USA). Continuous data were expressed as mean ± standard deviation (SD) and categorical data as frequencies (percentages). Comparisons between groups were conducted using the independent *t*−test or Mann–Whitney *U*-test for continuous variables and *χ*² test or Fisher’s exact test for categorical variables. Statistical significance was set at a two−tailed α < 0.05.

Univariate logistic regression analysis was performed to identify the candidate predictors of CAPO. Variables with a *P*−value <0.05 in the univariate analysis were considered for inclusion in the multivariable model. Prior to multivariable modeling, collinearity among candidate predictors was assessed using variance inflation factors. For pairs of variables exhibiting substantial collinearity (variance inflation factor >3) or conceptual redundancy—such as gestational age at diagnosis and preeclampsia onset, or systolic blood pressure and mean arterial pressure—the more clinically informative or continuous variable was retained.

Multivariable logistic regression with forward stepwise selection was then performed in the training cohort to identify the independent predictors of CAPO. The entry criterion was set at *P* <0.05 and the removal criterion at *P* >0.10. The results were expressed as odds ratios with corresponding 95% confidence intervals.

Two prediction models were constructed. Model A (the base model) was developed incorporating the independent predictors identified through the variable selection process described above, with the exception of the fibrinogen−to−albumin ratio. Model B (the extended model) was then constructed by adding the fibrinogen−to−albumin ratio to model A. The specific variables included in each model are reported in the “Results” section.

The performance of both models was evaluated in the training cohort, the internal validation cohort, and the external validation cohort. Discrimination was assessed using the area under the receiver operating characteristic curve. The DeLong test was used to compare the area under the curve of model A and model B. Calibration was evaluated with calibration plots and the Hosmer–Lemeshow goodness−of−fit test. Overall predictive accuracy was quantified using the Brier score.

The incremental prognostic value of adding FAR to model A was quantified in the entire cohort of 924 patients using the following metrics: the change in the area under the receiver operating characteristic curve, the continuous net reclassification improvement (category−free), and the integrated discrimination improvement.

Subgroup analyses were conducted in the training cohort to examine the consistency of the association between FAR and CAPO across prespecified strata, including preeclampsia onset (early−onset versus late−onset), preeclampsia severity (mild versus severe), fetal growth restriction (present versus absent), age (<35 versus ≥35 years), and pre−pregnancy body mass index (<28 versus ≥28 kg/m²). The interaction terms between FAR and each stratifying variable were tested in the multivariable model.

Sensitivity analyses were performed in the training cohort to evaluate the robustness of the findings under the alternative definitions of CAPO. These analyses included a restricted CAPO definition comprising only hard perinatal endpoints (placental abruption, neonatal respiratory distress syndrome, 5− min Apgar score <7, and perinatal death) and a definition that excluded elective preterm births.

## 3 Results

### 3.1 Patients’ characteristics and incidence of CAPO

A total of 1, 128 patients were initially screened, comprising 789 patients from center 1 and 339 from center 2. After application of the exclusion criteria, 680 eligible patients from center 1 were randomly assigned to either the training cohort (*n* = 476, 70%) or the internal validation cohort (*n* = 204, 30%). An additional 244 eligible patients from center 2 were designated as the external validation cohort (**Figure 1**). Within the training cohort, composite adverse perinatal outcome (CAPO) occurred in 199 of 476 patients, yielding an incidence of 41.8%. The baseline demographic, clinical, and laboratory characteristics of the training cohort, stratified by CAPO status, are summarized in **Table 1**. Patients who developed CAPO were older (32.90 ± 4.96 vs. 30.08 ± 4.97 years, *P* < 0.001) and had a higher pre−pregnancy body mass index (29.09 ± 3.92 vs. 26.49 ± 4.17 kg/m², *P* < 0.001) compared with those without CAPO. Gestational age at diagnosis was significantly earlier in the CAPO−positive group (33.43 ± 2.84 vs. 36.62 ± 2.52 weeks, *P* < 0.001), and systolic blood pressure at admission was markedly elevated (159.17 ± 11.96 vs. 140.98 ± 9.04 mmHg, *P* < 0.001). Correspondingly, early−onset preeclampsia (diagnosed before 34 weeks) and severe preeclampsia were more prevalent among patients with CAPO (50.25% vs. 25.99% and 58.79% vs. 13.72%, respectively; both *P* < 0.001). Fetal growth restriction was observed exclusively in the CAPO−positive group (32.16% vs. 0.00%, *P* < 0.001).

**Table 1:** Baseline of preeclamptic patients by CAPO status.

Variable	CAPO negative (n= 277)	CAPO positive (n=199)	P Value*
Demographics and clinical parameters at admission			
Age, years	30.08 ± 4.97	32.90 ± 4.96	<0.001
Pre-pregnancy BMI, kg/m²	26.49 ± 4.17	29.09 ± 3.92	<0.001
Gravidity, n (%)			0.550
1	59 (21.30)	44 (22.11)	
2	55 (19.86)	44 (22.11)	
3	49 (17.69)	40 (20.10)	
4	52 (18.77)	39 (19.60)	
5	62 (22.38)	32 (16.08)	
Nulliparous, n (%)	74 (26.71)	62 (31.16)	0.290
Previous preeclampsia, n (%)	17 (6.14)	13 (6.53)	0.861
Chronic hypertension, n (%)	17 (6.14)	15 (7.54)	0.547
Diabetes mellitus, n (%)	31 (11.19)	22 (11.06)	0.963
Smoking history, n (%)	16 (5.78)	4 (2.01)	0.043
Gestational age at diagnosis, weeks	36.62 ± 2.52	33.43 ± 2.84	<0.001
Systolic BP, mmHg	140.98 ± 9.04	159.17 ± 11.96	<0.001
Diastolic BP, mmHg	90.83 ± 6.80	91.51 ± 7.73	0.310
Mean arterial pressure, mmHg	108.84 ± 5.52	112.05 ± 6.70	<0.001
Preeclampsia characteristics			
Early-onset PE (<34 weeks), n (%)	72 (25.99)	100 (50.25)	<0.001
Severe PE, n (%)	38 (13.72)	117 (58.79)	<0.001
Fetal growth restriction, n (%)	0 (0.00)	64 (32.16)	<0.001
Baseline perfusion index (PI)	3.14 ± 1.11	3.23 ± 1.12	0.361
Laboratory findings at admission			
Fibrinogen, g/L	4.13 ± 0.75	4.47 ± 0.77	<0.001
Albumin, g/L	31.79 ± 4.00	31.44 ± 3.80	0.331
FAR	0.13 ± 0.03	0.14 ± 0.03	<0.001
Platelet count, ×10^9^/L	227.34 ± 46.71	162.03 ± 45.11	<0.001
Hemoglobin, g/L	116.04 ± 9.80	117.47 ± 10.80	0.134
Creatinine, μmol/L	57.79 ± 11.43	74.24 ± 12.83	<0.001
Uric acid, μmol/L	347.37 ± 63.17	343.82 ± 65.32	0.551
ALT, U/L	36.78 ± 19.98	51.74 ± 19.53	<0.001
AST, U/L	36.66 ± 16.11	35.01 ± 15.63	0.266
LDH, U/L	245.43 ± 58.82	249.76 ± 64.20	0.446
D-dimer, mg/L	1.68 ± 1.30	1.66 ± 1.15	0.863
sFlt-1/PlGF ratio	82.79 ± 30.69	159.69 ± 57.67	<0.001
Urine protein, g/24h	3.26 ± 2.18	2.88 ± 1.77	0.041
Perinatal outcomes and maternal complications			
Placental abruption, n (%)	0 (0.00)	27 (13.57)	<0.001
Preterm birth (<37 weeks), n (%)	0 (0.00)	188 (94.47)	<0.001
Fetal distress, n (%)	0 (0.00)	69 (34.67)	<0.001
NRDS, n (%)	0 (0.00)	41 (20.60)	<0.001
5-min Apgar <7, n (%)	0 (0.00)	42 (21.11)	<0.001
NICU admission, n (%)	0 (0.00)	97 (48.74)	<0.001
Perinatal death, n (%)	0 (0.00)	5 (2.51)	<0.001
Postpartum hemorrhage, n (%)	0 (0.00)	26 (13.07)	<0.001
Eclampsia, n (%)	0 (0.00)	11 (5.53)	<0.001
HELLP syndrome, n (%)	0 (0.00)	6 (3.02)	<0.001
Acute kidney injury, n (%)	0 (0.00)	6 (3.02)	<0.001

Data presented as mean ± SD, or n (%). P values from t-test, Wilcoxon rank-sum test, or chi-square test. BMI, body mass index; PE, preeclampsia; FAR, fibrinogen-to-albumin ratio; ALT, alanine aminotransferase; AST, aspartate aminotransferase; LDH, lactate dehydrogenase; CAPO, composite adverse perinatal outcome; NRDS, neonatal respiratory distress syndrome; NICU, neonatal intensive care unit.

With respect to laboratory findings, the CAPO−positive group exhibited a significantly higher fibrinogen−to−albumin ratio (FAR) than the CAPO−negative group (0.14 ± 0.03 vs. 0.13 ± 0.03, *P* < 0.001). In addition, patients with CAPO had lower platelet counts, higher creatinine concentrations, elevated alanine aminotransferase levels, and substantially higher sFlt−1/PlGF ratios (all *P* < 0.001). The distributions of gravidity, parity, previous preeclampsia, chronic hypertension, diabetes mellitus, and several other laboratory parameters did not differ significantly between the two groups. Among patients with CAPO, preterm birth occurred in 94.47%, NICU admission in 48.74%, fetal distress in 34.67%, and placental abruption in 13.57%. The incidences of eclampsia, HELLP syndrome, and acute kidney injury were 5.53%, 3.02%, and 3.02%, respectively.

Notably, patients with CAPO also had significantly lower platelet counts and higher FAR, both of which are relevant to anesthesia planning (e.g., neuraxial contraindication, bleeding risk).

### 3.2 Independent predictors of CAPO

Univariate logistic regression analysis was performed to identify the candidate predictors of CAPO. In the unadjusted analysis, the following variables were significantly associated with an increased risk of CAPO: age (odds ratio [OR] 1.120 per year, 95% confidence interval [CI] 1.077–1.164, *P* < 0.001), pre−pregnancy body mass index (OR 1.169 per kg/m², 95% CI 1.114–1.227, *P* < 0.001), early−onset preeclampsia (OR 2.876, 95% CI 1.954–4.233, *P* < 0.001), severe preeclampsia (OR 8.974, 95% CI 5.758–13.985, *P* < 0.001), systolic blood pressure (OR 1.168 per mmHg, 95% CI 1.136–1.200, *P* < 0.001), mean arterial pressure (OR 1.091 per mmHg, 95% CI 1.057–1.126, *P* < 0.001), FAR (OR 1.022, 95% CI 1.015–1.030, *P* < 0.001), platelet count (OR 0.970 per 10^9^/L, 95% CI 0.964–0.975, *P* < 0.001), creatinine (OR 1.117 per μmol/L, 95% CI 1.093–1.140, *P* < 0.001), alanine aminotransferase (OR 1.038 per U/L, 95% CI 1.027–1.048, *P* < 0.001), sFlt−1/PlGF ratio (OR 1.048 per unit, 95% CI 1.039–1.057, *P* < 0.001), and urine protein (OR 0.905 per g/24 h, 95% CI 0.822–0.997, *P* = 0.044). Gestational age at diagnosis was inversely associated with CAPO risk (OR 0.656 per week, 95% CI 0.603–0.713, *P* < 0.001). The remaining demographic, clinical, and laboratory variables did not reach statistical significance in the univariate analysis (**Table 2**).

**Table 2:** Univariate and multivariate logistic regression for CAPO predictors.

Variable	Univariate			Multivariate		
	OR	95% CI	P Value*	OR	95% CI	P Value*
Demographics and clinical parameters at admission						
Age, years	1.120	1.077-1.164	<0.001	1.332	1.172-1.513	<0.001
Pre-pregnancy BMI, kg/m²	1.169	1.114-1.227	<0.001	1.379	1.178-1.615	<0.001
Gravidity, n (%)						
1	REF		REF			
2	1.073	0.615-1.871	0.805			
3	1.095	0.618-1.939	0.757			
4	1.006	0.569-1.778	0.984			
5	0.692	0.388-1.234	0.212			
Nulliparous, n (%)	1.241	0.831-1.854	0.290			
Previous preeclampsia, n (%)	1.069	0.507-2.254	0.861			
Chronic hypertension, n (%)	1.247	0.607-2.56	0.548			
Diabetes mellitus, n (%)	0.986	0.553-1.761	0.963			
Smoking history, n (%)	0.335	0.11-1.017	0.053			
Gestational age at diagnosis, weeks	0.656	0.603-0.713	<0.001	0.359	0.256-0.503	<0.001
Systolic BP, mmHg	1.168	1.136-1.2	<0.001	1.205	1.123-1.292	<0.001
Diastolic BP, mmHg	1.013	0.988-1.039	0.309			
Mean arterial pressure, mmHg	1.091	1.057-1.126	<0.001			
Preeclampsia characteristics						
Early-onset PE (<34 weeks), n (%)	2.876	1.954-4.233	<0.001			
Severe PE, n (%)	8.974	5.758-13.985	<0.001			
Fetal growth restriction, n (%)	11.979	0.121-65.783	0.997			
Baseline perfusion index (PI)	1.079	0.916-1.271	0.360			
Laboratory findings at admission						
Fibrinogen, g/L	1.063	0.837-1.35	0.618			
Albumin, g/L	0.977	0.933-1.024	0.331			
FAR	1.022	1.015-1.030	<0.001	1.021	1.012-1.029	0.032
Platelet count, ×10^9^/L	0.970	0.964-0.975	<0.001			
Hemoglobin, g/L	1.014	0.996-1.032	0.134			
Creatinine, μmol/L	1.117	1.093-1.14	<0.001			
Uric acid, μmol/L	0.999	0.996-1.002	0.551			
ALT, U/L	1.038	1.027-1.048	<0.001	1.070	1.035-1.107	<0.001
AST, U/L	0.993	0.982-1.005	0.265			
LDH, U/L	1.001	0.998-1.004	0.445			
D-dimer, mg/L	0.987	0.851-1.144	0.863			
sFlt-1/PlGF ratio	1.048	1.039-1.057	<0.001	1.081	1.053-1.11	<0.001
Urine protein, g/24h	0.905	0.822-0.997	0.044			

Data presented as mean ± SD, or n (%). BMI, body mass index; PE, preeclampsia; FAR, fibrinogen-to-albumin ratio. Odds ratios for FAR are expressed per 0.01 unit increase to facilitate clinical interpretation, as the observed range of FAR in this cohort was 0.05–0.30; ALT, alanine aminotransferase; AST, aspartate aminotransferase; LDH, lactate dehydrogenase; CAPO, composite adverse perinatal outcome.

The variables that exhibited a univariate association with CAPO (*P* < 0.10) were considered for inclusion in the multivariable logistic regression model. Prior to model construction, collinearity among the candidate predictors was assessed using variance inflation factors (VIF). All variables retained in the final multivariable model demonstrated VIF values below 3.0, confirming the absence of substantial multicollinearity (**Supplementary Table S1**).

In the multivariable analysis, the following factors were identified as independent predictors of CAPO: age (adjusted OR 1.332 per year, 95% CI 1.172–1.513, *P* < 0.001), pre−pregnancy body mass index (adjusted OR 1.379 per kg/m², 95% CI 1.178–1.615, *P* < 0.001), gestational age at diagnosis (adjusted OR 0.359 per week, 95% CI 0.256–0.503, *P* < 0.001), systolic blood pressure (adjusted OR 1.205 per mmHg, 95% CI 1.123–1.292, *P* < 0.001), alanine aminotransferase (adjusted OR 1.070 per U/L, 95% CI 1.035–1.107, *P* < 0.001), and the sFlt−1/PlGF ratio (adjusted OR 1.081 per unit, 95% CI 1.053–1.110, *P* < 0.001). Notably, FAR remained an independent predictor after multivariable adjustment (adjusted OR 1.021, 95% CI 1.012–1.029, *P* = 0.032). The full univariate and multivariable regression results are presented in **Table 2**.

### 3.3 Model development and validation

Two multivariable logistic regression models were constructed using the training cohort (*n* = 476). Model A incorporated the independent predictors identified in the multivariable analysis, namely, age, pre−pregnancy body mass index, gestational age at diagnosis, systolic blood pressure, platelet count, creatinine, alanine aminotransferase, and the sFlt−1/PlGF ratio. Model B included all variables from model A with the addition of FAR.

**Figure 2:**
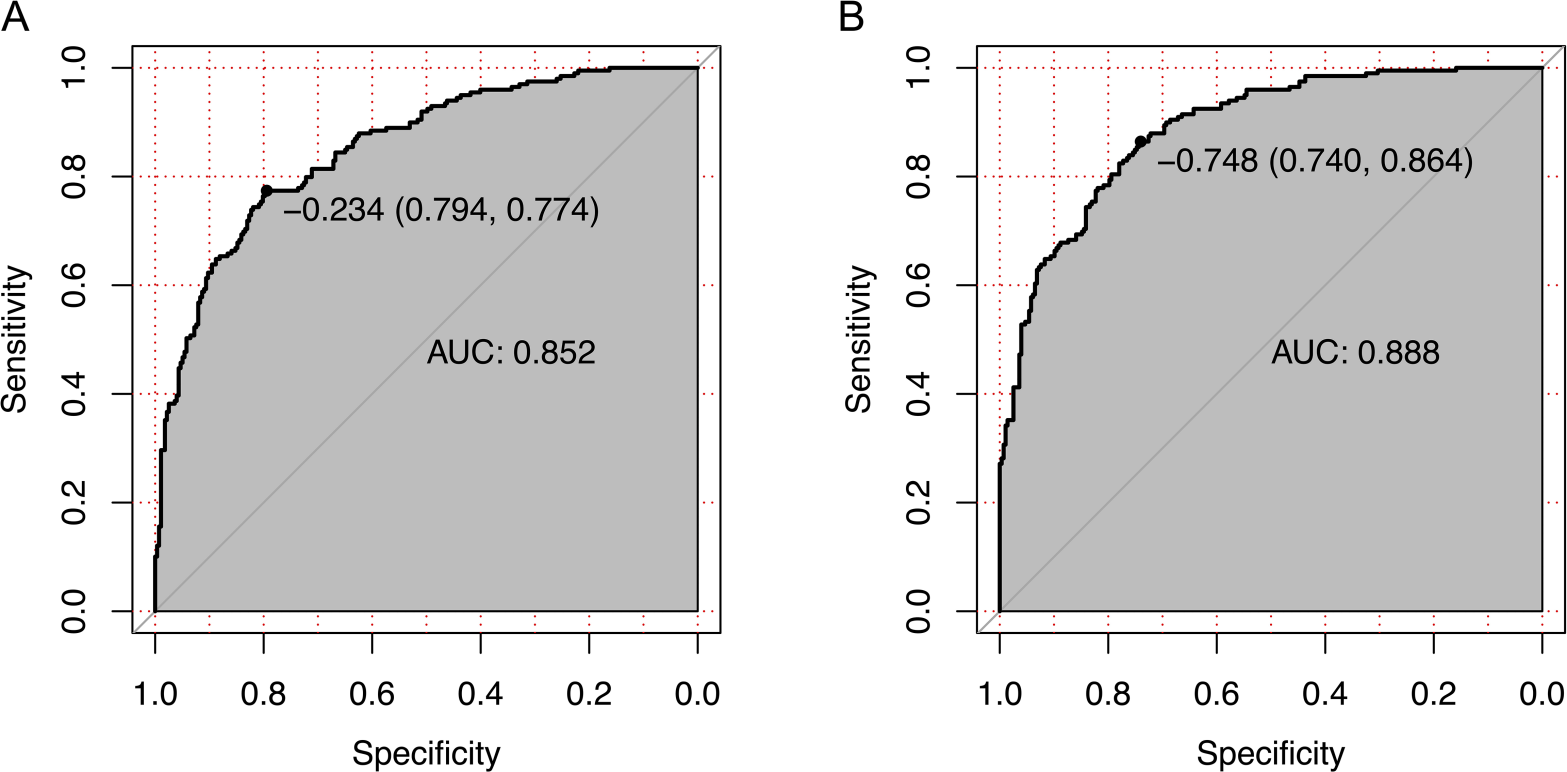
Receiver operating characteristic (ROC) curves for predicting composite adverse perinatal outcome in the training cohort. The area under the ROC curve (AUC) is shown for model A (base model) and model B (base model plus fibrinogen−to−albumin ratio). The diagonal dashed line represents an AUC of 0.50, indicating no discriminative ability. AUC, area under the curve.

**Figure 3:**
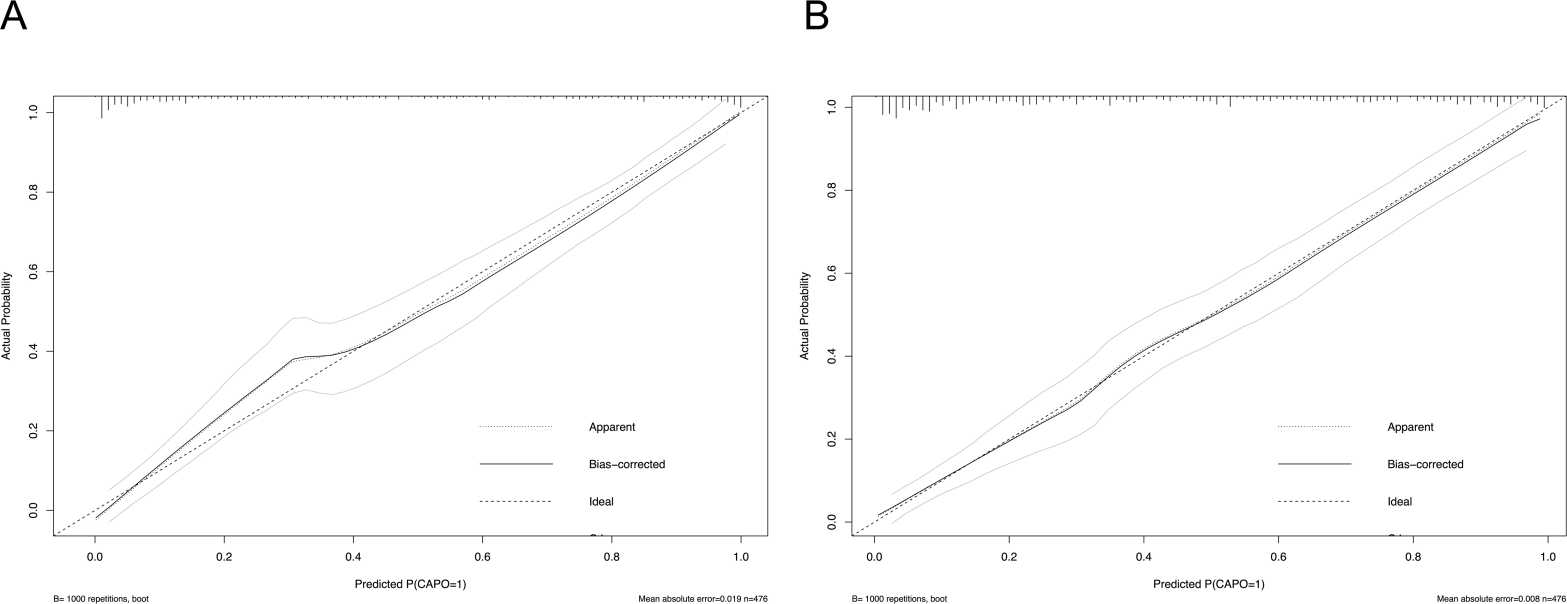
Calibration curves for model A and model B in the training cohort. Calibration was assessed by plotting the predicted probability against the observed probability of composite adverse perinatal outcome. The dashed diagonal line represents perfect calibration. The solid line depicts the bias−corrected calibration curve obtained from 1, 000 bootstrap resamples. The distribution of predicted probabilities is shown by the rug plot along the x−axis.

In the training cohort, model A demonstrated good discrimination for CAPO, with an area under the ROC curve (AUC) of 0.852 (95% confidence interval [CI] 0.818–0.887). The inclusion of FAR in model B resulted in a statistically significant improvement in discrimination, yielding an AUC of 0.888 (95% CI 0.859–0.917; *P* < 0.001 for DeLong test; **Figure 2**). The optimal cutoff value for FAR was 0.135, which corresponded to a sensitivity of 0.864 (95% CI 0.814–0.910) and a specificity of 0.740 (95% CI 0.689–0.791), respectively. Both models exhibited satisfactory calibration in the training set, as reflected by Hosmer–Lemeshow *P*−values of 0.691 for model A and 0.594 for model B and Brier scores of 0.153 and 0.134, respectively (**Figure 3**).

The performance of both models was further assessed in the internal validation cohort (*n* = 204). Model B maintained excellent discrimination, with an AUC of 0.893 (95% CI 0.850–0.937), which was significantly higher than that of model A (AUC 0.877, 95% CI 0.831–0.923; *P* = 0.011). The Brier score for model B in the internal validation set was 0.129, indicating good overall predictive accuracy.

To evaluate generalizability, the models were applied to an external validation cohort from a second center (*n* = 244). Model B achieved an AUC of 0.856 (95% CI 0.808–0.904), again surpassing model A (AUC 0.798, 95% CI 0.740–0.857; *P* = 0.003). The Brier score for model B in the external cohort was 0.145. It is worth noting that the addition of FAR was associated with a marked increase in sensitivity in the external validation cohort (from 0.698 to 0.860), albeit with a modest reduction in specificity (from 0.759 to 0.728).

### 3.4 Incremental value of FAR

The incremental prognostic value of adding FAR to model A was assessed in the entire cohort of 924 patients. The addition of FAR was associated with a modest but statistically significant improvement in discrimination, as reflected by an increase in the area under the receiver operating characteristic curve (ΔAUC) of 0.010 (95% confidence interval [CI] 0.002–0.019; *P* = 0.013). Furthermore, the extended model demonstrated substantial net reclassification improvement, with a continuous net reclassification index (NRI) of 0.397 (95% CI 0.268–0.526; *P* < 0.001). The integrated discrimination improvement (IDI) was 0.022 (95% CI 0.012–0.032; *P* = 0.004) (**Table 3**).

**Table 3:** Performance comparison and incremental value of FAR for predicting CAPO.

Metric	Model A (Base model)	Model B (Base model + FAR)	P Value*
Training set (n=476)			
AUC (95% CI)	0.852(0.818-0.887)	0.888 (0.859-0.917)	<0.001¹
Sensitivity²	0.774(0.719-0.829)	0.864 (0.814-0.910)	
Specificity²	0.794(0.744-0.838)	0.740 (0.689-0.791)	
Brier score	0.153	0.134	
Hosmer-Lemeshow χ²	5.606	6.481	
Hosmer-Lemeshow P	0.691	0.594	
Internal validation set (n=204)			
AUC (95% CI)	0.877(0.831-0.923)	0.893(0.850-0.937)	0.011¹
Sensitivity²	0.753(0.659-0.835)	0.788(0.694 0.871)	
Specificity²	0.849(0.782-0.908)	0.857(0.790-0.916)	
Brier score	0.140	0.129	
External validation cohort (n=244)			
AUC (95% CI)	0.798(0.740-0.857)	0.856(0.808-0.904)	0.003¹
Sensitivity²	0.698(0.605-0.791)	0.860(0.790-0.930)	
Specificity²	0.759(0.690- 0.823)	0.728(0.658-0.797)	
Brier score	0.173	0.145	
Incremental value (entire cohort, n=924)			
ΔAUC (95% CI)	REF	0.010(0.002--0.019)	0.013¹
NRI (continuous)³ (95% CI)	REF	0.397 (0.268–0.526)	<0.001
IDI (95% CI)	REF	0.022 (0.012–0.032)	0.004

Data are presented as point estimate (95% confidence interval) unless otherwise indicated.

AUC, area under the receiver operating characteristic curve; NRI, net reclassification improvement; IDI, integrated discrimination improvement.

¹P-value from DeLong test for comparison of AUCs.

²Sensitivity and specificity calculated at the optimal cut-off determined by Youden index in the training set.

³Continuous NRI (category-free) calculated without predefined risk thresholds.

Model A (Base model): included age, pre-pregnancy BMI, gestational age at diagnosis, systolic blood pressure, platelet count, creatinine, ALT, and sFlt-1/PlGF ratio.

Model B (Extended model): included all variables in Model A plus FAR (fibrinogen-to-albumin ratio).

### 3.5 Nomogram construction

**Figure 4:**
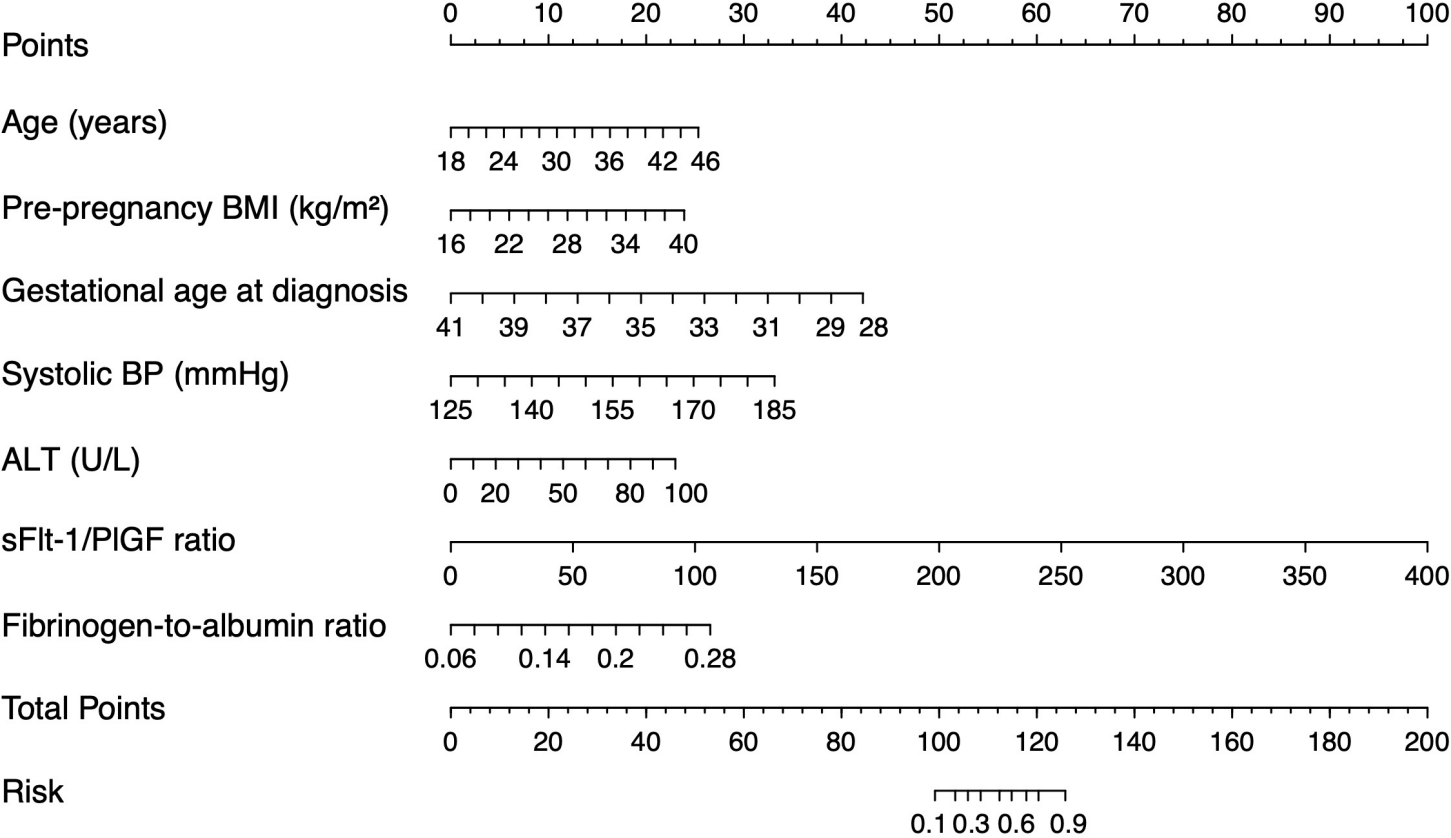
Nomogram for predicting composite adverse perinatal outcome based on the extended model (model B). To estimate the probability of CAPO, locate each predictor value on its respective axis, draw a vertical line upward to the “Points “ axis to determine the assigned score, and sum the points for all predictors. The total points correspond to the predicted risk on the “Risk “ axis at the bottom of the nomogram. BMI, body mass index; BP, blood pressure; ALT, alanine aminotransferase; FAR, fibrinogen−to−albumin ratio.

To facilitate individualized risk estimation in clinical practice, a nomogram was constructed based on the extended multivariable model (model B). The nomogram incorporated the following independent predictors: age, pre−pregnancy body mass index, gestational age at diagnosis, systolic blood pressure, alanine aminotransferase, the sFlt−1/PlGF ratio, and the fibrinogen−to−albumin ratio. Each predictor was assigned a weighted point value corresponding to its regression coefficient. The total points accumulated across all predictors correspond to the estimated probability of CAPO. The nomogram is presented in **Figure 4**.

### 3.6 Clinical utility

**Figure 5:**
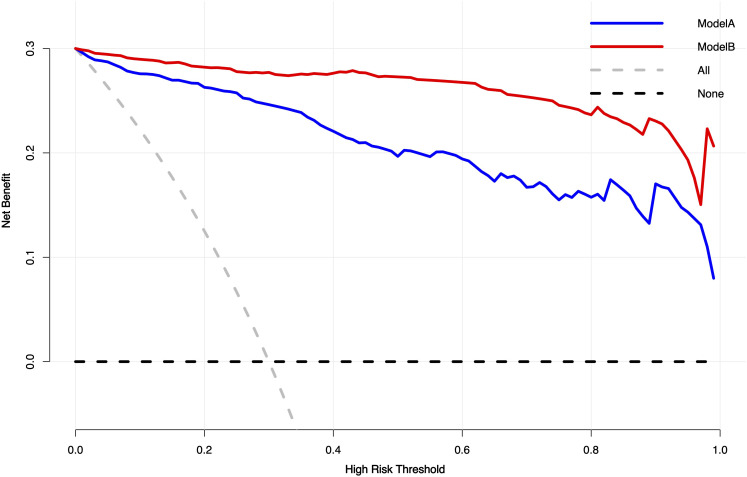
Decision curve analysis of model **(A)** and model **(B)** for predicting composite adverse perinatal outcome. The net benefit is plotted against threshold probability. Model B (blue curve) demonstrated a consistently higher net benefit compared with model **(A)** (red curve) across a range of clinically relevant threshold probabilities. The gray curve represents the strategy of considering all patients as high−risk (treat−all), and the black curve represents the strategy of considering no patients as high−risk (treat−none).

The clinical utility of model B was evaluated using decision curve analysis and compared with that of model A (**Figure 5**). Across a wide range of clinically relevant threshold probabilities, model B demonstrated a modest but consistently higher net benefit than model A. Although the greatest net benefit for both models was observed at very low threshold values, such thresholds would necessitate intervention in nearly all patients and are therefore clinically impractical. As the risk threshold increased, the net benefit of both models gradually declined, yet model B maintained a favorable advantage over model A throughout the range of 0.10 to 0.50.

**Figure 6:**
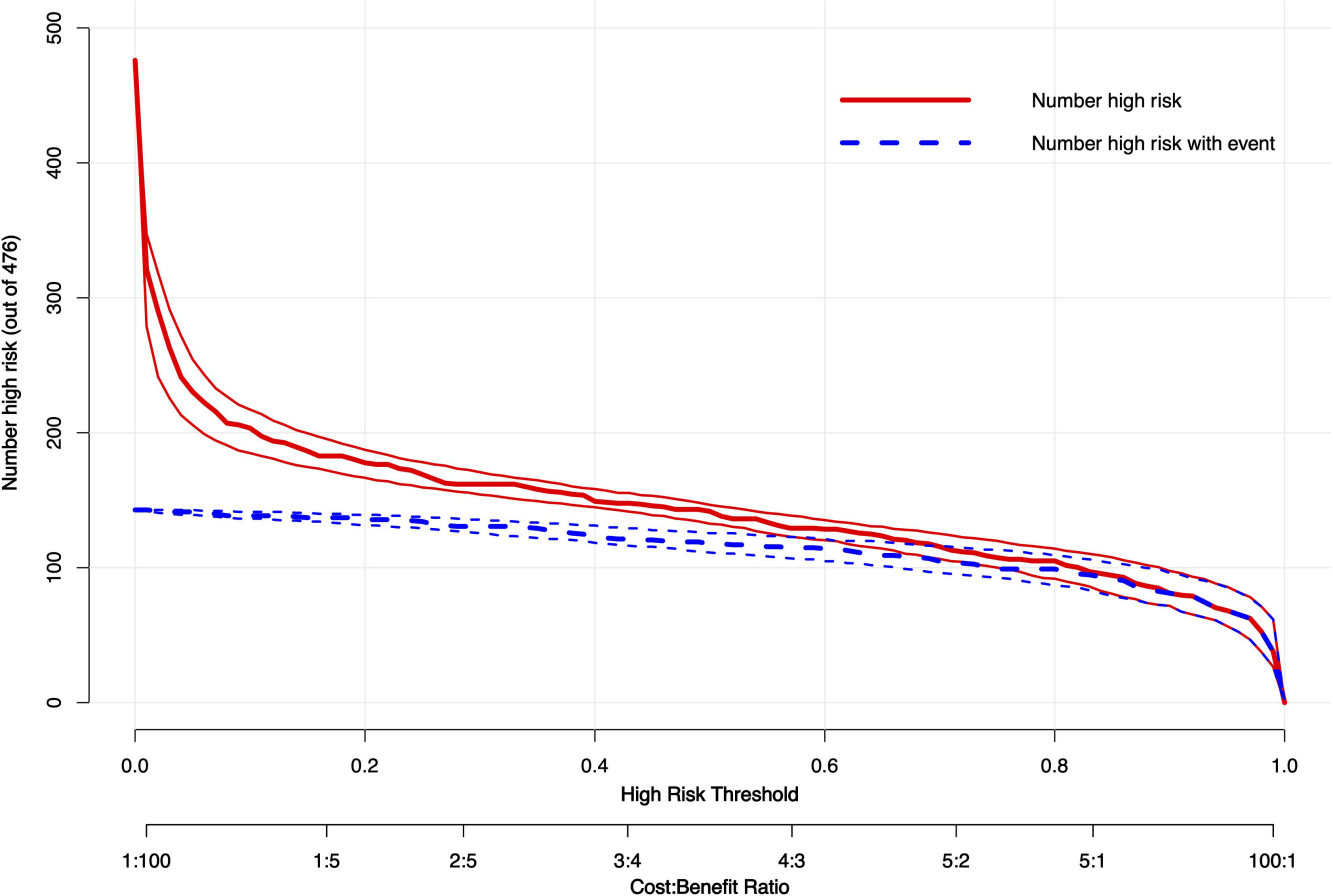
Clinical impact curve of the extended model (model B) in the training cohort. The red curve depicts the number of patients classified as high−risk at each threshold probability. The blue curve shows the number of true positive cases (patients who actually experienced CAPO) among those classified as high−risk. The plot illustrates the trade−off between the proportion of the cohort identified as high−risk and the proportion of adverse events captured.

The clinical impact curve (**Figure 6**) illustrated the trade−off between the number of patients classified as high−risk and the proportion of true CAPO events captured at varying threshold probabilities. At a threshold of 0.28, a total of 178 patients (37.4% of the training cohort) were classified as high−risk. Among these high−risk classifications, 82.6% of all actual CAPO events were captured, while false−positive classifications accounted for 17.4% of the high−risk group. This threshold corresponded to a net benefit of 0.28 in the decision curve analysis and reflected a favorable balance between event detection and intervention specificity. Lower thresholds (e.g., <0.15) were associated with a marked increase in false−positive classifications without a proportional gain in net benefit.

### 3.7 Subgroup analyses

**Figure 7:**
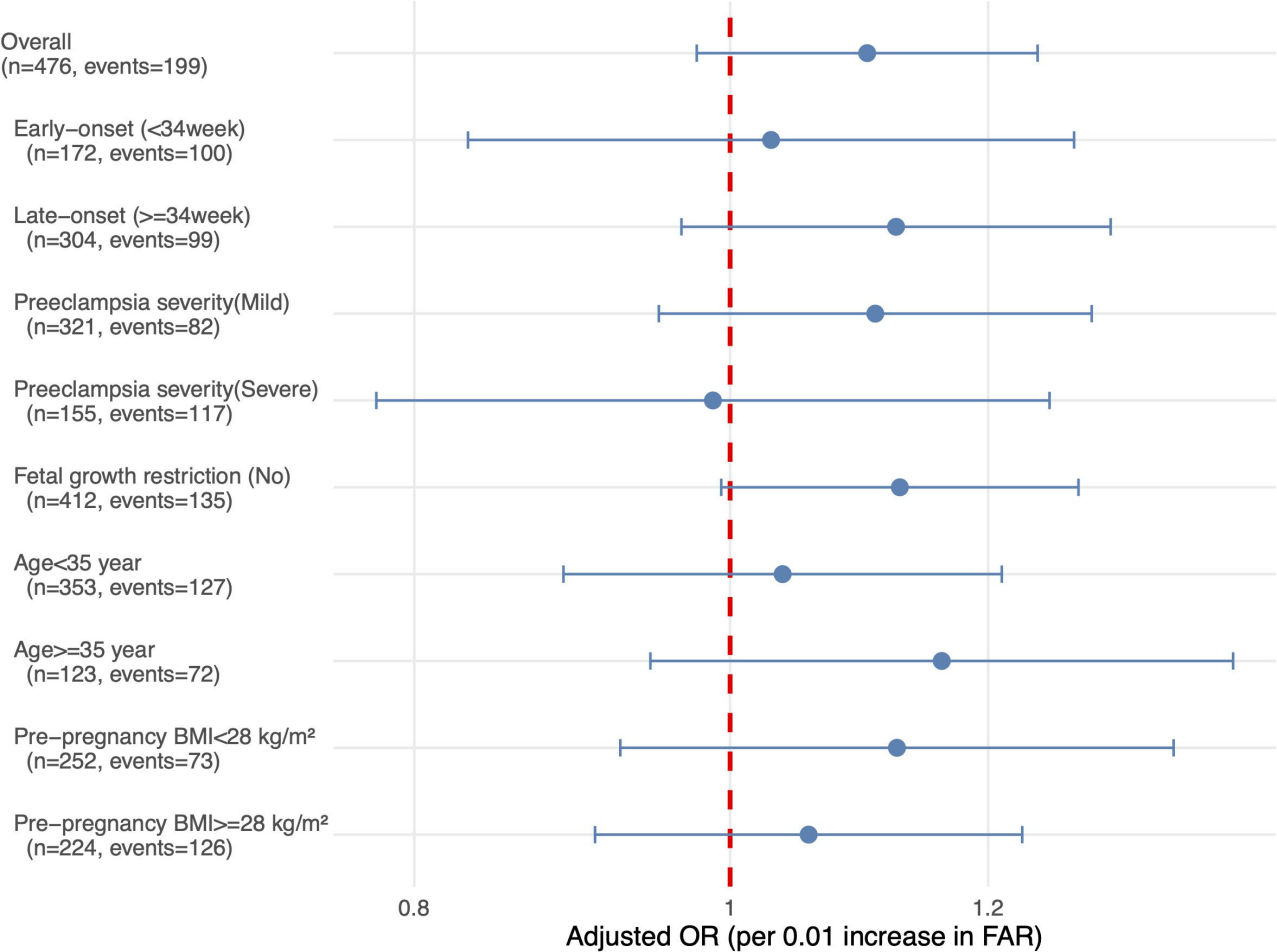
Forest plot of subgroup analyses evaluating the association between fibrinogen−to−albumin ratio and composite adverse perinatal outcome in the training cohort. Adjusted odds ratios (per 0.01 increase in FAR) and 95% confidence intervals are displayed for the overall cohort and for prespecified subgroups. The vertical dashed line represents an odds ratio of 1.0 (no association). *P*−values for interaction were derived from multivariable logistic regression models, including an interaction term. OR, odds ratio; CI, confidence interval; BMI, body mass index.

Subgroup analyses were performed in the training cohort to assess the consistency of the association between FAR and CAPO across predefined clinical strata (**Supplementary Table S2**; **Figure 7**). The direction of the association between FAR and CAPO was consistently positive across all evaluated subgroups, and no statistically significant interactions were observed. Specifically, the association did not differ meaningfully according to preeclampsia onset (early−onset: adjusted OR 1.029, 95% CI 0.831–1.275; late−onset: adjusted OR 1.124, 95% CI 0.966–1.308; *P* for interaction = 0.425) or preeclampsia severity (mild: adjusted OR 1.108, 95% CI 0.951–1.291; severe: adjusted OR 0.988, 95% CI 0.779–1.253; *P* for interaction = 0.681). Similarly, the association was consistent across strata defined by age (*P* for interaction = 0.696) and pre−pregnancy body mass index (*P* for interaction = 0.494). In patients without fetal growth restriction, the adjusted OR was 1.127 (95% CI 0.994–1.279). Among patients with fetal growth restriction, the OR was not estimable due to complete separation, as all individuals in this subgroup experienced CAPO by definition.

### 3.8 Sensitivity analyses

Sensitivity analyses were conducted in the training cohort to evaluate the robustness of the association between FAR and CAPO under alternative outcome definitions (**Supplementary Table S3**). Under the primary definition, which included all eight components of CAPO, the adjusted odds ratio per 0.01 increase in FAR was 1.102 (95% confidence interval [CI] 1.067–1.143; *P* = 0.039). When the outcome was restricted to hard perinatal endpoints—namely, placental abruption, neonatal respiratory distress syndrome, 5−min Apgar score below 7, and perinatal death—the association remained statistically significant, with an adjusted odds ratio of 1.073 (95% CI 1.039–1.124; *P* = 0.045). After the exclusion of patients with elective preterm birth, the point estimate for FAR remained in the same direction, although the association was attenuated and no longer statistically significant (adjusted OR 1.052, 95% CI 0.927–1.194; *P* = 0.430). This attenuation likely reflects both a reduction in sample size (from 476 to 423) and the removal of a key pathway through which FAR may influence adverse outcomes.

## 4 Discussion

This dual−center retrospective cohort study demonstrated that the fibrinogen−to−albumin ratio provides independent and incremental prognostic value for predicting composite adverse perinatal outcome in patients with established preeclampsia, beyond the model A that already incorporated the sFlt−1/PlGF ratio and other conventional clinical and laboratory predictors. The addition of FAR resulted in a statistically significant improvement in discrimination (ΔAUC 0.010, 95% CI 0.002–0.019; *P* = 0.013), a substantial net reclassification improvement of approximately 40% (continuous NRI 0.397, 95% CI 0.268–0.526; *P* < 0.001), and a modest but significant integrated discrimination improvement (IDI 0.022, 95% CI 0.012–0.032; *P* = 0.004). The FAR−enhanced model maintained robust performance in both internal and external validation cohorts and exhibited favorable clinical utility across a range of decision thresholds.

The association between FAR and adverse perinatal outcomes observed in this study is biologically plausible. Preeclampsia is characterized by systemic inflammation, endothelial dysfunction, and activation of the coagulation cascade (37, 38). Fibrinogen, an acute−phase reactant, rises in response to interleukin−6−mediated inflammation, whereas albumin declines due to reduced hepatic synthesis and increased capillary permeability (39). FAR therefore integrates these two opposing signals into a single index that reflects the net burden of inflammation and coagulopathy. This mechanistic rationale is supported by recent studies demonstrating that FAR is associated with disease severity in preeclampsia and with adverse perinatal events (33, 34, 40–42). The present study extends these observations by showing that the prognostic information conveyed by FAR is not fully captured by the sFlt−1/PlGF ratio or by routine clinical parameters, suggesting that FAR reflects pathophysiological pathways that are at least partially distinct from angiogenic imbalance.

Several aspects of the incremental value analysis merit a detailed discussion. The ΔAUC of 0.010 was statistically significant but numerically modest. This is not unexpected, as model A already incorporated powerful predictors including gestational age, systolic blood pressure, platelet count, and the sFlt−1/PlGF ratio. In such a well−performing baseline model, any single additional biomarker is unlikely to produce a large absolute increase in discrimination. More importantly, the continuous NRI of 0.397 indicates that FAR correctly reclassifies approximately 40% of patients into more appropriate risk categories. To contextualize this finding, an NRI exceeding 0.2 is generally considered potentially informative in cardiovascular and obstetric prediction research (43). An NRI of 0.397 means that for every 100 preeclamptic patients evaluated, the addition of FAR would correctly the reassign risk status for approximately 40 individuals—a magnitude of improvement that could meaningfully influence decisions regarding the intensity of maternal–fetal monitoring, timing of delivery, and level of neonatal care (33, 44). The IDI of 0.022, although numerically small, was statistically significant and further supports the conclusion that FAR enhances the overall discriminative capacity of the model.

The present findings should be interpreted in the context of existing prediction models for preeclampsia. The fullPIERS model, developed from a large prospective multicenter cohort, is widely used to predict adverse maternal outcomes within 48 h to 7 days after admission (19–21). However, a recent validation study reported that the fullPIERS model had limited performance in predicting perinatal outcomes, with an AUC of only 0.561 (95% CI 0.480–0.642, *P* = 0.642) (19). This limitation underscores the need for prognostic tools that specifically encompass fetal and neonatal endpoints. The PREP−L model was designed specifically for women with early−onset preeclampsia and focuses on maternal complications by discharge (45). The present study addresses these gaps by developing a model specifically tailored to predict CAPO—a composite endpoint encompassing both maternal and perinatal complications—in a broader population of preeclamptic patients, regardless of gestational age at diagnosis.

The findings of this study also extend the existing literature on FAR in preeclampsia. Zorlu and colleagues first reported that a higher FAR was associated with adverse perinatal events, including placental abruption (OR 2.1, 95% CI 1.1–3.9), neonatal respiratory distress syndrome (OR 2.25, 95% CI 1.1–4.4), and NICU admission (OR 2.19, 95% CI 1.1–4.2) (46). However, their study had a relatively small sample size of 112 preeclamptic patients and did not evaluate whether FAR provides incremental value beyond established biomarkers such as the sFlt−1/PlGF ratio (47). A larger Chinese study involving 3, 249 participants demonstrated that FAR was independently associated with preeclampsia (OR 6.16, 95% CI 3.82–9.94) but that its standalone discriminatory capacity was modest (AUC approximately 0.60), suggesting that FAR is best utilized in combination with other clinical and laboratory parameters (2). The present study confirms and extends these observations by demonstrating, in a well−characterized cohort of 924 patients with rigorous external validation, that FAR adds meaningful incremental prognostic information even when added to a model that already includes the sFlt−1/PlGF ratio.

Beyond its prognostic utility, FAR offers several practical advantages that facilitate clinical implementation. Unlike the sFlt−1/PlGF ratio, which requires specialized immunoassay platforms and is not routinely available in many low− and middle−income settings, FAR is derived from two inexpensive and universally available laboratory tests—fibrinogen and albumin—that are already included in standard coagulation and liver function panels for preeclamptic patients. This widespread availability, combined with minimal incremental cost, makes FAR a particularly attractive adjunct for risk stratification in resource−constrained environments where advanced biomarker testing may be inaccessible. This allows anesthesiologists to calculate FAR within minutes at the bedside using routine admission labs, without waiting for specialized angiogenic assays.

The subgroup analyses provided additional reassurance regarding the robustness of the FAR–CAPO association. The direction of the effect was consistently positive across strata defined by preeclampsia onset, disease severity, fetal growth restriction, age, and pre−pregnancy body mass index. No statistically significant interactions were detected, indicating that the prognostic value of FAR is not materially modified by these clinical characteristics. The consistent effect observed across both early−onset and late−onset preeclampsia is particularly noteworthy, as these subtypes are thought to have distinct pathophysiological mechanisms. This finding supports the generalizability of FAR as a prognostic marker across the full spectrum of preeclamptic disease.

The sensitivity analyses further confirmed the robustness of the primary findings. When the outcome definition was restricted to hard perinatal endpoints—namely, placental abruption, neonatal respiratory distress syndrome, 5− min Apgar score below 7, and perinatal death—FAR remained a statistically significant predictor (adjusted OR 1.073, 95% CI 1.039–1.124; *P* = 0.045). This addresses the potential concern that the primary composite outcome might be influenced by more subjective components such as fetal distress or elective preterm birth. After the exclusion of elective preterm births, the point estimate for FAR remained in the same direction, although statistical significance was lost (adjusted OR 1.052, 95% CI 0.927–1.194; *P* = 0.430). This attenuation is likely attributable to a combination of reduced sample size (from 476 to 423) and the removal of a key mechanistic pathway: FAR may predict the development of severe diseases that necessitates indicated preterm delivery. Thus, the loss of significance in this sensitivity analysis does not undermine the overall conclusion but rather provides insight into the potential mechanisms through which FAR exerts its prognostic effect.

The clinical utility of the FAR−enhanced model was evaluated using decision curve analysis. Across a broad range of clinically relevant threshold probabilities (0.10 to 0.50), model B demonstrated consistently higher net benefit than Model A. The clinical impact curve further illustrated that at a threshold of 0.28, the model identified approximately 37% of patients as high−risk while capturing over 82% of actual CAPO events. This balance between event detection and resource utilization is clinically attractive, as it allows for targeted intensification of monitoring without overwhelming healthcare resources. Such a strategy could facilitate more efficient allocation of perioperative monitoring resources and guide anesthesia planning for high−risk patients.

The findings of this study have several practical implications for anesthesiologists managing preeclamptic patients. First, FAR can be calculated rapidly from routine coagulation and liver function tests. This calculation requires no additional cost or specialized equipment. Second, a FAR value exceeding 0.135 (the optimal cutoff identified in this study) may serve as a warning sign of heightened coagulopathy and systemic inflammation. In such patients, neuraxial anesthesia should be considered with caution. Platelet count and clotting profile should be reviewed before proceeding. Third, an elevated FAR may help identify patients at increased risk of hemodynamic instability and postpartum hemorrhage. The early establishment of invasive arterial monitoring and central venous access should be considered. Preparedness for massive transfusion is also advisable in these patients. Fourth, the nomogram developed in this study (**Figure 4**) provides a bedside tool for anesthesiologists to estimate individual CAPO risk. This risk estimate can be integrated into preoperative discussions with obstetricians and neonatologists. Finally, patients with a high FAR and a high predicted CAPO risk may benefit from planned admission to a higher−acuity setting after delivery, such as an intensive care unit or a high−dependency unit. These potential benefits, however, should be interpreted in light of the study’s limitations.

Several limitations of this study should be acknowledged. First, the retrospective design carries inherent risks of selection bias and residual confounding, although rigorous inclusion criteria and comprehensive multivariable adjustment were employed to mitigate these concerns. Second, the study was conducted in two tertiary referral centers in China, and external validation in more diverse populations and healthcare settings is warranted before widespread clinical implementation. Third, the sFlt−1/PlGF ratio was measured using a single commercial assay platform, and the generalizability of the FAR−enhanced model to other assay systems remains to be established. Fourth, the absence of long−term follow−up data precludes assessment of the relationship between FAR and maternal cardiovascular outcomes or offspring neurodevelopment.

Future prospective studies are needed to validate the FAR−enhanced prediction model in broader populations and to assess its impact on clinical decision−making and patient outcomes. In particular, implementation studies evaluating whether FAR−guided risk stratification can reduce adverse perinatal events or optimize resource allocation would be of considerable clinical interest. Second, the optimal threshold of FAR for clinical decision−making remains to be established. While the present study identified a cutoff of 0.135 based on the Youden index, this threshold requires prospective validation in independent cohorts and may vary across populations with different baseline FAR distributions. Third, whether serial FAR measurements during the antepartum period provide additional prognostic information beyond a single admission value warrants investigation. Given that both fibrinogen and albumin concentrations may fluctuate with disease progression, longitudinal FAR trajectories could offer dynamic risk stratification and guide the timing of intervention.

Pending further prospective validation, clinicians may consider incorporating FAR into the initial risk assessment of preeclamptic patients. A FAR value exceeding 0.135, particularly when accompanied by other high−risk features such as early−onset disease, severe hypertension, or a markedly elevated sFlt−1/PlGF ratio, should prompt heightened vigilance, more frequent maternal–fetal monitoring, and early consultation with neonatal intensive care teams. Conversely, a low FAR in a clinically stable patients may provide reassurance and support a more conservative management approach. The nomogram developed in this study provides a practical tool for integrating FAR with other established predictors to generate individualized risk estimates at the bedside.

## 5 Conclusions

In conclusion, this dual−center retrospective cohort study demonstrates that FAR provides statistically significant and potentially useful incremental prognostic value for predicting CAPO in patients with established preeclampsia, beyond the model A that includes the sFlt−1/PlGF ratio and other established predictors. The FAR−enhanced model exhibited robust discrimination and calibration in internal and external validation cohorts and showed favorable clinical utility. For anesthesiologists, FAR−guided risk assessment may facilitate more informed decisions regarding neuraxial versus general anesthesia, invasive monitoring, blood product preparedness, and postoperative care intensity.

## Data Availability

The original contributions presented in the study are included in the article/**Supplementary Material**. Further inquiries can be directed to the corresponding authors.
