# Supplementary material for: Incremental prognostic value of the fibrinogen−to−albumin ratio for adverse perinatal outcomes in preeclampsia: a dual−center retrospective cohort study
Source: Front Endocrinol (Lausanne). 2026 May 29;17:1853375. doi: 10.3389/fendo.2026.1853375 (PMC13259769; doi:10.3389/fendo.2026.1853375)
Supplement: Supplementary file 1 [file Table1.docx]

| Supplementary Table 1. Variance inflation factors for variables in the final multivariable model | |
| --- | --- |
|  |  |
| Variable | VIF |
|  |  |
| Age, years | 1.08 |
| Pre-pregnancy BMI, kg/m² | 1.07 |
| Gestational age at diagnosis, weeks | 1.16 |
| Systolic BP, mmHg | 1.43 |
| FAR | 1.16 |
| Platelet count, ×10⁹/L | 1.06 |
| Creatinine, μmol/L | 1.22 |
| ALT, U/L | 1.12 |
| sFlt-1/PlGF ratio | 1.45 |
| VIF = variance inflation factor. All VIF values <3.0, indicating no significant multicollinearity. | |
