# Supplementary material for: Incremental prognostic value of the fibrinogen−to−albumin ratio for adverse perinatal outcomes in preeclampsia: a dual−center retrospective cohort study
Source: Front Endocrinol (Lausanne). 2026 May 29;17:1853375. doi: 10.3389/fendo.2026.1853375 (PMC13259769; doi:10.3389/fendo.2026.1853375)
Supplement: Supplementary file 2 [file Table2.docx]

| Supplementary Table 2. Subgroup analyses of the association between FAR and CAPO in the training set | | | | |
| --- | --- | --- | --- | --- |
|  |  |  |  |  |
| Subgroup | n | CAPO events (%) | Adjusted OR (95% CI) | P for interaction |
|  |  |  |  |  |
| Overall | 476 | 199 (41.8%) | 1.102 (0.977-1.243) |  |
| Preeclampsia onset |  |  |  | 0.425 |
| <34week | 172 | 100 (58.1%) | 1.029 (0.831-1.275) |  |
| ≥34week | 304 | 99 (32.6%) | 1.124 (0.966-1.308) |  |
| Preeclampsia severity |  |  |  | 0.681 |
| Mild | 321 | 82 (25.5%) | 1.108 (0.951-1.291) |  |
| Severe | 155 | 117 (75.5%) | 0.988 (0.779-1.253) |  |
| Fetal growth restriction |  |  |  | 1 |
| No | 412 | 135 (32.8%) | 1.127 (0.994-1.279) |  |
| Yes | 64 | 64 (100%) | NE |  |
| Age |  |  |  | 0.696 |
| <35 year | 353 | 127 (36%) | 1.038 (0.889-1.212) |  |
| ≥35 year | 123 | 72 (58.5%) | 1.161 (0.945-1.427) |  |
| Pre-pregnancy BMI |  |  |  | 0.494 |
| <28 kg/m2 | 252 | 73 (29%) | 1.125 (0.925-1.368) |  |
| ≥28 kg/m2 | 224 | 126 (56.2%) | 1.057 (0.909-1.229) |  |
| Adjusted OR per 0.01 increase in FAR, adjusted for age, pre-pregnancy BMI, gestational age at diagnosis, systolic BP, platelet count, creatinine, ALT, and sFlt-1/PlGF ratio (except when the variable is the stratifying variable).Interaction P-value for FGR subgroup should be interpreted with caution as CAPO event rate is 100% in the FGR group by definition (FGR is a component of CAPO). NE = Not estimable due to complete separation (all patients in this subgroup had CAPO event | | | | |
|  |  |  |  |  |
|  |  |  |  |  |
|  |  |  |  |  |
