# Supplementary material for: Incremental prognostic value of the fibrinogen−to−albumin ratio for adverse perinatal outcomes in preeclampsia: a dual−center retrospective cohort study
Source: Front Endocrinol (Lausanne). 2026 May 29;17:1853375. doi: 10.3389/fendo.2026.1853375 (PMC13259769; doi:10.3389/fendo.2026.1853375)
Supplement: Supplementary file 3 [file Table3.docx]

| Supplementary Table 3. Sensitivity Analyses for the Association Between FAR and CAPO Under Alternative Outcome Definitions | | | | |
| --- | --- | --- | --- | --- |
|  |  |  |  |  |
| CAPO definition | n | Events (%) | Adjusted OR (95% CI) | P-value |
|  |  |  |  |  |
| Primary definition (all 8 components) | 476 | 199 (41.8%) | 1.102 (1.067-1.143) | 0.039 |
| Restricted CAPO (hard perinatal outcomes only) | 476 | 95 (20%) | 1.073 (1.039-1.124) | 0.045 |
| Excluding elective preterm birth | 423 | 146 (34.5%) | 1.052 (0.927-1.194) | 0.430 |
| Data are from the training set unless otherwise specified.Adjusted OR per 0.01 increase in FAR, adjusted for age, pre-pregnancy BMI, gestational age at diagnosis, systolic BP, platelet count, creatinine, ALT, and sFlt-1/PlGF ratio.Primary CAPO includes: placental abruption, preterm birth (<37 weeks), fetal growth restriction, fetal distress, NRDS, 5-min Apgar <7, NICU admission, and perinatal death.Restricted CAPO includes: placental abruption, NRDS, 5-min Apgar <7, and perinatal death. Elective preterm birth defined as medically indicated delivery before 37 weeks without spontaneous labor or rupture of membranes. | | | | |
|  |  |  |  |  |
|  |  |  |  |  |
|  |  |  |  |  |
